# Rational engineering of *Halomonas salifodinae* to enhance hydroxyectoine production under lower-salt conditions

**DOI:** 10.1007/s00253-024-13197-0

**Published:** 2024-05-31

**Authors:** Niping Yang, Mengshuang Liu, Jing Han, Mingyue Jiang, Yan Zeng, Ying Liu, Hua Xiang, Yanning Zheng

**Affiliations:** 1 State Key Laboratory of Microbial Resources, Institute of Microbiology, Chinese Academy of Sciences, No.1 Beichen West Road, Chaoyang District, Beijing, 100101 China; 2 School of Life Sciences, Hebei University, Baoding, 071002 China; 3 College of Life Science, University of Chinese Academy of Sciences, Beijing, 100049 China

**Keywords:** Hydroxyectoine, *Halomonas*, Metabolic engineering, Lower-salt fermentation, High-value chemical

## Abstract

**Abstract:**

Hydroxyectoine is an important compatible solute that holds potential for development into a high-value chemical with broad applications. However, the traditional high-salt fermentation for hydroxyectoine production presents challenges in treating the high-salt wastewater. Here, we report the rational engineering of *Halomonas salifodinae* to improve the bioproduction of hydroxyectoine under lower-salt conditions. The comparative transcriptomic analysis suggested that the increased expression of *ectD* gene encoding ectoine hydroxylase (EctD) and the decreased expressions of genes responsible for tricarboxylic acid (TCA) cycle contributed to the increased hydroxyectoine production in *H. salifodinae* IM328 grown under high-salt conditions. By blocking the degradation pathway of ectoine and hydroxyectoine, enhancing the expression of *ectD*, and increasing the supply of 2-oxoglutarate, the engineered *H. salifodinae* strain HS328-YNP15 (Δ*doeA*::P_UP119_-*ectD* p-*gdh*) produced 8.3-fold higher hydroxyectoine production than the wild-type strain and finally achieved a hydroxyectoine titer of 4.9 g/L in fed-batch fermentation without any detailed process optimization. This study shows the potential to integrate hydroxyectoine production into open unsterile fermentation process that operates under low-salinity and high-alkalinity conditions, paving the way for next-generation industrial biotechnology.

**Key points:**

• *Hydroxyectoine production in H. salifodinae correlates with the salinity of medium*

• *Transcriptomic analysis reveals the limiting factors for hydroxyectoine production*

• *The engineered strain produced 8.3-fold more hydroxyectoine than the wild type*

**Graphical Abstract:**

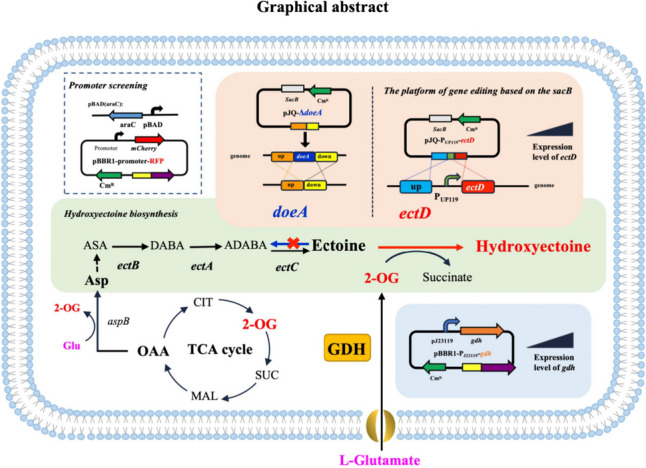

**Supplementary Information:**

The online version contains supplementary material available at 10.1007/s00253-024-13197-0.

## Introduction

Halophilic bacteria enable themselves to survive in high salt and alkaline environments by importing or biosynthesizing a variety of compatible solutes (Kempf and Bremer 1998). Hydroxyectoine is such a crucial compatible solute that helps halophilic bacteria maintain turgor pressure without interfering with normal cellular processes (Brown 1976; Inbar and Lapidot 1988). Compared with its biosynthetic precursor ectoine that binds seven water molecules, hydroxyectoine that can bind up to nine water molecules possesses a stronger hydration property. Besides, hydroxyectoine possesses excellent protective properties that can effectively enhance the protein stabilization, offering a promising reagent to protect proteins against a variety of harmful environmental stresses such as high salinity, heat, desiccation, freezing, and radiation (Lippert and Galinski 1992; Pastor et al. 2010; Tanne et al. 2014). Beyond its bioprotective role, hydroxyectoine has the potential to be developed into drugs in treating diseases such as Alzheimer’s and rhinoconjunctivitis (Bilstein et al. 2021; Kanapathipillai et al. 2005; Salapatek et al. 2011). Therefore, the biosynthesis of hydroxyectoine has attracted increasing attention due to its promising prospects across various applications (Graf et al. 2008; Liu et al. 2021).

**Fig. 1:**
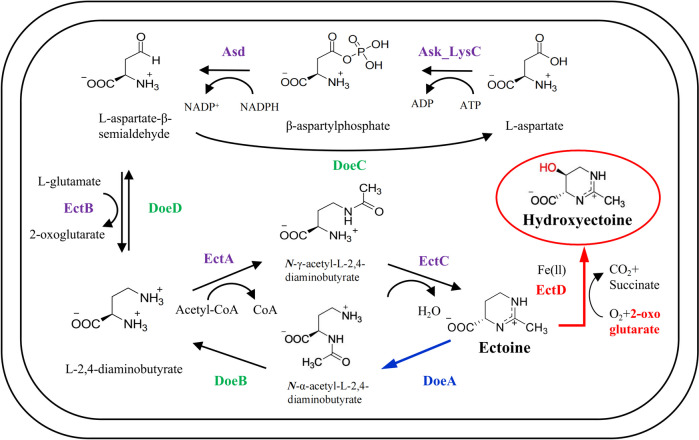
Biosynthesis and biodegradation pathways of hydroxyectoine (Kunte et al. 2014). The hydroxyectoine biosynthesis starts with the conversion of aspartate to ectoine sequentially catalyzed by L-aspartate kinase (Ask_LysC), L-aspartate-β-semialdehyde dehydrogenase (Asd), L-2,4-diaminobutyrate aminotransferase (EctB), L-2,4-diaminobutyrate acetyltransferase (EctA), and ectoine synthase (EctC). Finally, ectoine is converted to hydroxyectoine in a 2-oxoglutarate-requiring reaction catalyzed by ectoine hydroxylase (EctD). The degradation of ectoine/hydroxyectoine is proposed to begin with the opening of the pyrimidine ring in a reaction catalyzed by ectoine hydrolase DoeA

The biosynthesis of hydroxyectoine begins with aspartate serving as the initial precursor (Fig. 1). The L-aspartate kinase (Ask_LysC) and L-aspartate-β-semialdehyde dehydrogenase (Asd) are responsible for the conversion of aspartate to aspartate-β-semialdehyde. Subsequently, a three-step enzymatic reaction involving L-2,4-diaminobutyrate aminotransferase (EctB), L-2,4-diaminobutyrate acetyltransferase (EctA), and ectoine synthase (EctC) catalyzes the synthesis of ectoine from aspartate-β-semialdehyde (Louis and Galinski 1997; Ono et al. 1999). The conversion of ectoine to hydroxyectoine is conducted by ectoine hydroxylase (EctD), a member of the non-heme-containing iron (II) and 2-oxoglutarate-dependent dioxygenase superfamily (Bursy et al. 2007; Garcia-Estepa et al. 2006; Reuter et al. 2010; Widderich et al. 2014b).

In the process of ectoine and hydroxyectoine production known as “bacterial milking,” halophilic bacteria such as *Halomonas elongata* are first grown under high-salt conditions to accumulate ectoine and hydroxyectoine (Sauer and Galinski 1998). Subsequently, these bacterial cells are transferred to water to release ectoine and hydroxyectoine. However, the accumulation of hydroxyectoine is positively correlated with the salinity of fermentation broth, causing the traditional high-salt fermentation to face huge challenges in the treatment of high-salt wastewater (Liu et al. 2021). Therefore, many attempts have been made to engineer non-halophilic bacteria for the production of hydroxyectoine under lower-salt conditions. By expressing the *ectABCD_ask* gene cluster from *Pseudomonas stutzeri* A1501 in *E. coli* FF4169, which cannot synthesize the compatible solute trehalose, 14.39 mg/g DCW hydroxyectoine was obtained in the presence of 0.3 mol/L NaCl, with an almost equivalent amount of ectoine produced (Stoeveken et al. 2011). When a single *ectD* gene from *P. stutzeri* A1501 was expressed in *E. coli* FF4169, 2.13 g/L ectoine supplemented in the medium was completely converted into hydroxyectoine within 24 h at a salinity of 0.4 mol/L NaCl (Czech et al. 2016). In a similar experiment, the *ectABCD-ask* gene cluster from *P. stutzeri* DSM5190^T^ was introduced into *E. coli* DH5α, resulting in the predominant production of hydroxyectoine (over 95%) at a yield of 79.08 mg/g DCW in low-salt conditions (0.34 mol/L NaCl) (Seip et al. 2011). Additionally, the engineered *E. coli* expressing the hydroxyectoine biosynthetic pathway from *Acidiphilium cryptum* produced 1.6 g/L of hydroxyectoine at 0.08 mol/L NaCl, with a yield of 2.2 g/g DCW (Bethlehem and Moritz 2020). By optimizing the heterologous expression of hydroxyectoine biosynthetic genes and controlling the intracellular 2-oxoglutarate levels through quorum sensing-based autoregulation, an engineered *E. coli* employing an efficient osmolarity-independent production strategy achieved hydroxyectoine production of 14.93 g/L, with a yield as high as 1.678 g/g DCW (Ma et al. 2022).

Besides *E. coli*, an engineered *Hansenula polymorpha* expressing the codon-optimized *ectABCD* genes from *Halomonas elongata* produced hydroxyectoine with a yield of 57.73 mg/g dry cell weight (DCW) in the absence of NaCl (Eilert et al. 2013). Recently, hydroxyectoine production from carbon dioxide (CO_2_)-based biorefineries has been demonstrated in halotolerant hydrogen-oxidizing bacteria that naturally have the hydroxyectoine biosynthetic genes, with *Rhodococcus opacus* and *Hydrogenibacillus schlegelii* achieving yields of 53 mg/g biomass and 62 mg/g biomass at salinities of 1.20 and 0.85 mol/L NaCl, respectively (Cantera et al. 2023; Liang et al. 2024; Liu et al. 2023). Hybrid biological-inorganic systems were also employed for the production of hydroxyectoine from CO_2_ in halotolerant hydrogen-oxidizing *Achromobacter xylosoxidans* and *Mycolicibacterium mageritense*, which produced 208.77 µg/L and 9.49 µg/L hydroxyectoine at a salinity equivalent to seawater, respectively (Feng et al. 2023). In addition, by heterologously expressing of codon-optimized *ectD* from *P. stutzeri* A1501, the recombinant *Corynebacterium glutamicum* produced 74 g/L hydroxyectoine from ectoine, representing the highest hydroxyectoine titer reported so far (Jungmann et al. 2022). However, these processes still require high temperature sterilization, as these chassis cells are unable to survive in extreme conditions that can effectively prevent the growth of unwanted microorganisms. To decrease the energy consumption and production cost of hydroxyectoine production, it is necessary to develop a new strategy for efficient bioproduction of hydroxyectoine under lower-salt but higher-alkali conditions that allow open unsterile fermentation (Rodriguez-Moya et al. 2013).

Here, we explored the influence of salinity on the biosynthesis of hydroxyectoine by comparative transcriptomic analysis of halo- and alkali-tolerant *Halomonas salifodinae* IM328 grown under low-salt and high-salt conditions, respectively, and found that the expression levels of *ectD* and hydroxyectoine degradative pathway and the availability of 2-oxoglutarate affected the hydroxyectoine biosynthesis. Moreover, metabolic engineering of *H. salifodinae* IM328 was carried out to improve the hydroxyectoine production under lower-salt but alkaline conditions, and the finally obtained *H. salifodinae* strain HS328-YNP15 (Δ*doeA*::P_UP119_-*ectD* p-*gdh*) finally achieved an 8.3-fold higher hydroxyectoine production compared to the wild-type strain, showing its potential for future hydroxyectoine production into open unsterile fermentation processes under conditions of low salinity and high alkalinity.

## Materials and methods

### Strains, plasmids, and growth conditions

The strains and plasmids used in this study are listed in Table 1. *H. salifodinae* IM328 (CGMCC 22183) grown in 80-LB medium was used as the start strain for genetic engineering (Zhang et al. 2021), while *E. coli* S17-1 grown in LB medium (containing 0.2 mol/L NaCl) was used for the plasmid constructions and conjugations (Fu et al. 2014). The 80-LB medium is the same as LB medium but contains 1.4 mol/L NaCl (Ma et al. 2020). For shake-flask culture, all *H. salifodinae* and *E. coli* strains were grown at 37 °C in an orbital shaker at 200 rpm.

**Table 1:** Strains and plasmids used in this work

Strain or plasmid	Genotype or phenotype	Source
Strains		
* E. coli* S17-1	*pro hdsR recA*; chromosomal insertion of RP4-2 (Tc::Mu *K*_*m*_::Tn7)	(Simon et al. 1983)
* Halomonas salifodinae* IM328	Wild type	(Zhang et al. 2021)
HS328-YNP01	*H. salifodinae* IM328 harboring pBBR1-P_J23100_-RFP	This work
HS328-YNP02	*H. salifodinae* IM328 harboring pBBR1-P_J23110_-RFP	This work
HS328-YNP03	*H. salifodinae* IM328 harboring pBBR1-P_J23111_-RFP	This work
HS328-YNP04	*H. salifodinae* IM328 harboring pBBR1-P_J23119_-RFP	This work
HS328-YNP05	*H. salifodinae* IM328 harboring pBBR1-P_UP119_-RFP	This work
HS328-YNP06	*H. salifodinae* IM328 harboring pBBR1-P_Tac_-RFP	This work
HS328-YNP07	*H. salifodinae* IM328 harboring pBBR1-P_BAD_-RFP	This work
HS328-YNP08	*H. salifodinae* IM328 harboring pBBR1-P_BAD_-*ectD*	This work
HS328-YNP09	*H. salifodinae* IM328 harboring pBBR1-P_J23110_-*ectD*	This work
HS328-YNP10	*H. salifodinae* IM328 harboring pBBR1-P_J23119_-*ectD*	This work
HS328-YNP11	*H. salifodinae* IM328 harboring pBBR1-P_UP119_-*ectD*	This work
HS328-YNP12	*H. salifodinae* IM328 Δ*doeA*	This work
HS328-YNP13	P_UP119_ promoter was introduced into the HS328-YNP12 genome before its *ectD* gene using allelic exchange	This work
HS328-YNP14	HS328-YNP12 harboring pBBR1-P_J23119_-*gdh*	This work
HS328-YNP15	HS328-YNP13 harboring pBBR1-P_J23119_-*gdh*	This work
Plasmids		
pBBR-rfp-ptac	Cm^R^ and Sm^R^, a derivative plasmid of pBBR1MCS	(Mu et al. 2017)
pBBR1-P_Tac_	Cm^R^ and Sm^R^; *MCS-TrrnB* cloned into the *Hin*dIII/*Bam*HI site of pBBR1-rfp-ptac, *MCS-TrrnB* was amplified from pBBR1-rfp-ptac	This work
pBBR1-P_Tac_-RFP	Cm^R^ and Sm^R^; *mCherry* gene (GenBank: PP001104) cloned into the *Hin*dIII/*Nde*I site of pBBR1-P_Tac_	This work
pBBR1-P_J23100_-RFP	Cm^R^ and Sm^R^; P_J23100_ promoter cloned into *Xho*I/*Hin*dIII site of pBBR1-P_Tac_-RFP	This work
pBBR1-P_J23110_-RFP	Cm^R^ and Sm^R^; P_J23110_ promoter cloned into *Xho*I/*Hin*dIII site of pBBR1-P_Tac_-RFP	This work
pBBR1-P_J23111_-RFP	Cm^R^ and Sm^R^; P_J23111_ promoter cloned into *Xho*I/*Hin*dIII site of pBBR1-P_Tac_-RFP	This work
pBBR1-P_J23119_-RFP	Cm^R^ and Sm^R^; P_J23119_ promoter cloned into *Xho*I/*Hin*dIII site of pBBR1-P_Tac_-RFP	This work
pBBR1-P_UP119_-RFP	Cm^R^ and Sm^R^; P_UP119_ promoter cloned into *Xho*I/*Hin*dIII site of pBBR1-P_Tac_-RFP	This work
pBBR1-P_BAD_-RFP	Cm^R^ and Sm^R^; P_BAD(*araC*)_ promoter cloned into *Xho*I/*Hin*dIII site of pBBR1-P_Tac_-RFP	This work
pBBR1-*ectD*	Cm^R^ and Sm^R^; *ectD* gene cloned into *Xho*I/*Nde*I site of pBBR1-P_Tac_	This work
pBBR1-P_BAD_-*ectD*	Cm^R^ and Sm^R^; P_BAD(*araC*)_ promoter cloned into *Xho*I site of pBBR1-*ectD*	This work
pBBR1-P_J23110_-*ectD*	Cm^R^ and Sm^R^; P_J23110_ promoter cloned into *Xho*I site of pBBR1-*ectD*	This work
pBBR1-P_J23119_-*ectD*	Cm^R^ and Sm^R^; P_J23119_ promoter cloned into *Xho*I site of pBBR1-*ectD*	This work
pBBR1-P_UP119_-*ectD*	Cm^R^ and Sm^R^; P_UP119_ promoter cloned into *Xho*I site of pBBR1-*ectD*	This work
pBBR1-P_J23119_-*gdh*	Cm^R^ and Sm^R^; *gdh* cloned into *Hin*dIII/*Nde*I site of pBBR1-P_J23119_-RFP	This work
pJQ200SK	Gm^R^; *sacB*; mobilizable suicide vector	(Quandt and Hynes 1993)
pJQ	Cm^R^; the same as the pJQ200SK but harbors chloramphenicol resistance gene instead of gentamicin resistance gene	This work
pJQ-Δ*doeA*	Cm^R^; in-frame Δ*doeA* cloned into *Xho*I/*Bam*HI site of pJQ	This work
pJQ-P_UP119_-*ectD*	Cm^R^; in-frame P_UP119_ cloned into *Xho*I site of pJQ	This work

### Total RNA preparation and comparative transcriptomic analysis

*H. salifodinae* IM328 (GenBank: JADOTW000000000.1) was grown at 37 °C in LB medium containing 0.2 and 3.0 mol/L NaCl, respectively. After the cells reached the log phase of growth, 10 mL cultures were harvested and centrifuged at 4 °C for 10 min. The resultant pellets were then quickly frozen using liquid nitrogen before being stored at −80 °C. Total RNA extraction and transcriptome sequencing were carried out by Shanghai Majorbio Bio-Pharm Technology Co., Ltd. The RSEM (http://deweylab.github.io/RSEM/) and the DESeq2 (http://bioconductor.org/packages/release/bioc/html/DESeq2.html) software packages were employed to quantify the gene expression and identify the differentially expressed genes (DEGs), respectively. To account for multiple testing, the false discovery rate (FDR) was calculated to adjust the threshold of the *p *value. DEGs were defined as genes with FDR values < 0.01 and |log_2_ (fold change)| >1 (Liu et al. 2018). The Gene Ontology (GO) enrichment analysis of DEGs was conducted using Goatools (https://github.com/tanghaibao/GOatools). KEGG, as a database resource, was used to interpret the higher-order functional meanings and utilities of the organisms based on molecular-level information (Kanehisa et al. 2004). Consequently, KEGG pathway enrichment analysis was carried out to assess statistical significance.

### Genetic manipulation of *H. salifodinae*

The pBBR1-P_Tac_-RFP plasmid was constructed as follows. The *MCS-TrrnB* DNA fragment amplified from pBBR-rfp-ptac (Mu et al. 2017) by PCR using the Q5 high-fidelity DNA polymerase (New England Biolabs) was inserted into the *Hin*dIII/*Bam*HI-digested pBBR-rfp-ptac using the T5 exonuclease-dependent assembly system (Xia et al. 2019) to obtain pBBR1-P_Tac_, which was then digested by *Hin*dIII and *Nde*I and ligated with the *mCherry* gene (GenBank: PP001104) to obtain the pBBR1-P_Tac_-RFP plasmid. To examine the strengths of different promoters, the P_UP119_, P_J23119_, P_J23111_, P_J23110_, P_J23100_, and P_BAD(*araC*)_ promoters were incorporated into the *Xho*I/*Hin*dIII-digested pBBR1-P_Tac_-RFP respectively. The constructed pBBR1-prompter-RFP plasmids were mobilized into *H. salifodinae* IM328 respectively by conjugation with *E. coli* S17-1 to obtain different *H. salifodinae* strains (HS328-YNP01 to HS328-YNP07) (Zhang et al. 2021). The *ectD* gene (GenBank: JADOTW010000016.1 /IHD13_09630) coding for ectoine hydroxylase in the genome of *H. salifodinae* IM328 was amplified and inserted into *Xho*I/*Nde*I-digested pBBR1-P_Tac_ to obtain the pBBR1-*ectD* plasmid. Then, P_UP119_, P_J23119_, P_J23110_, and P_BAD (*araC*)_ promoters were incorporated into the *Xho*I-digested pBBR1-*ectD* for the plasmid-based overexpression of *ectD*. The constructed pBBR1-prompter-*ectD* plasmids were mobilized into *H. salifodinae* IM328 respectively by conjugation with *E. coli* S17-1 to obtain different *H. salifodinae* strains (HS328-YNP08 to HS328-YNP11) (Zhang et al. 2021).

In-frame deletion of *doeA* gene (GenBank: JADOTW010000003.1/IHD13_02420) from the ectoine degradation pathway (Schwibbert et al. 2011) was achieved as follows. Q5 high-fidelity DNA polymerase was used to amplify 1189 bp of DNA upstream of its start codon and 1184 bp of DNA downstream of its stop codon by PCR. Using the T5 exonuclease-dependent assembly system, the two DNA fragments obtained by PCR were incorporated into *Xho*I/*Bam*HI-digested pJQ suicide plasmid. This plasmid is the same as the pJQ200SK suicide plasmid except that it harbors chloramphenicol resistance gene in place of gentamicin resistance gene. The obtained pJQ-Δ*doeA* plasmid was then mobilized into *H. salifodinae* IM328 by conjugation with *E. coli* S17-1. Double-crossover event for allelic exchange was achieved using a selection and screening strategy employed in *Rhodopseudomonas palustris* (Rey et al. 2007). The *H. salifodinae* Δ*doeA* mutant (strain HS328-YNP12), whose *doeA* gene was deleted in the genome, was finally obtained.

For the overexpression of *ectD* gene coding for ectoine hydroxylase, substitution of the native promoter in front of *ectD* with P_UP119_ promoter in the genome of HS328-YNP12 was achieved by using a pJQ suicide vector. Q5 high-fidelity DNA polymerase was used to amplify 1073 bp of DNA upstream of P*ectD* promoter, 84 bp of P_UP119_ promoter DNA, and 936 bp of DNA downstream of P*ectD* promoter by PCR. These three DNA fragments were incorporated into pJQ suicide vector using the T5 exonuclease-dependent assembly system to obtain the plasmid pJQ-P_UP119_-*ectD*. The HS328-YNP12 strain was used as the parent strain and pJQ-P_UP119_-*ectD* was mobilized into HS328-YNP12 by conjugation, using the same protocol as mentioned above. Double-crossover event for allelic exchange was achieved using a selection and screening strategy employed in *Rhodopseudomonas palustris* (Rey et al. 2007) to obtain *H. salifodinae* Δ*doeA*::P_UP119_-*ectD* (strain HS328-YNP13).

For the overexpression of glutamate dehydrogenase, the genomic DNA of *H. elongata* DSM2581 (CGMCC 1.6329) was first used as template to amplify *gdh* gene (GenBank: FN869568.2/ HELO_3049) by PCR. The PCR fragment of *gdh* gene was then incorporated into the *Hin*dIII/*Nde*I-digested pBBR1-P_J23119_-RFP to obtain pBBR1-P_J23119_-*gdh* plasmid, which was then mobilized into HS328-YNP12 and HS328-YNP13 by conjugation with *E. coli* S17-1 to obtain the *H. salifodinae* Δ*doeA* p-*gdh* (strain HS328-YNP14) and *H. salifodinae* Δ*doeA*::P_UP119_-*ectD* p-*gdh* (strain HS328-YNP15), respectively (Zhang et al. 2021). The mutations of all engineered strains were verified through PCR and sequencing. Primers used in this study are listed in Supplementary Table S1.

### Measurement of the fluorescence intensity

The cell pellets of the recombinant *H. salifodinae* strains (HS328-YNP01 to HS328-YNP07) overexpressing *mCherry* gene were washed three times and then resuspended with 10 mM phosphate-buffered saline (PBS). Then, 200 µL of cell suspension solution was transferred to a 96-well black microplate for the measurement of relative fluorescence intensity by a microplate reader (Synergy TMH4, BioTek Instruments), with the excitation and emission wavelengths configured to 587 nm and 610 nm, respectively (Jiang et al. 2022).

### Shake-flask and fed-batch fermentation

For shake-flask culture, *H. salifodinae* strains (HS328-YNP08 to HS328-YNP15) were grown in MME or MMD medium under different salt conditions (37 °C, 200 rpm). The MME medium consisted of 30 g/L glucose, 1 g/L yeast extract, 0.25 g/L (NH_4_)_2_SO_4_, 0.2 g/L MgSO_4_, 9.65 g/L Na_2_HPO_4_·12H_2_O, 1.5 g/L KH_2_PO_4_, 1.7 g/L citric acid, 17.71 g/L sodium aspartate, 7.46 g/L KCl, 9 g/L beef extract, 10 mL/L trace element solution I, 1 mL/L trace element solution II, and varied amounts of NaCl. The trace element solution I contained 5 g/L Fe(III)-NH_4_-citrate, 2 g/L CaCl_2_, and 1 M HCl, while the trace element solution II comprised 100 mg/L ZnSO_4_·7H_2_O, 30 mg/L MnCl_2_·4H_2_O, 300 mg/L H_3_BO_3_, 200 mg/L CoCl_2_·6H_2_O, 10 mg/L CuSO_4_·5H_2_O, 20 mg/L NiCl_2_·6H_2_O, and 30 mg/L NaMoO_4_·2H_2_O. The pH of the medium was adjusted to 8.0 using 5 M NaOH (Ma et al. 2020). The MMD medium is identical to the MME medium, with the sole distinction being the substitution of sodium aspartate with sodium glutamate.

A 5-L bioreactor (Baoxing Co., China) containing 2 L of MMD medium supplemented with 1.4 mol/L NaCl was used for fed-batch culture of the finally obtained *H. salifodinae* Δ*doeA*::P_UP119_-*ectD* p-*gdh* (strain HS328-YNP15). The dissolved oxygen (DO%) was maintained at ~ 30% of air saturation by flushing air with a maximum flow rate of 1 VVM (air volume per culture volume per min). The OD_600_, the concentration of glucose, sodium glutamate, ectoine, and hydroxyectoine were determined every 4 h.

### Ectoine and hydroxyectoine measurements

To quantify the extracellular ectoine and hydroxyectoine, cultures were centrifuged at 12,000 rpm at room temperature for 2 min to collect their supernatants, which were then subjected to a 20-fold dilution for HPLC analysis. To measure the amounts of intracellular ectoine and hydroxyectoine, the cell pellets were first resuspended with 1 mL of ultrapure water and then treated with three freeze-thaw cycles (− 80 °C for 15 min and 65 °C for 2 min). The cell lysates were centrifuged at 12,000 rpm at room temperature for 20 min to collect the supernatants for HPLC analysis. Agilent 1260 Infinity II system equipped with an Inertsil NH_2_ column (4.6 × 250 mm, 5 μm, GL Sciences, Tokyo, Japan) was used to measure the ectoine and hydroxyectoine. Acetonitrile/ultrapure water (70:30, v/v) was used as the mobile phase at a flow rate of 1 mL/min. The detection wavelength of 210 nm was used to measure the ectoine and hydroxyectoine. The standard curves of ectoine and hydroxyectoine were created by determining the HPLC response to the commercially available ectoine (Mreda Technology Co., Beijing, China) and hydroxyectoine (Macklin Biochemical Technology Co., Shanghai, China) at a range of concentrations (0.0, 0.2, 0.4, 0.6, 0.8, and 1.0 g/L), respectively, with absorbance on the *y*-axis and concentration on the *x*-axis.

## Results

### The effect of salinity on hydroxyectoine production

**Fig. 2:**
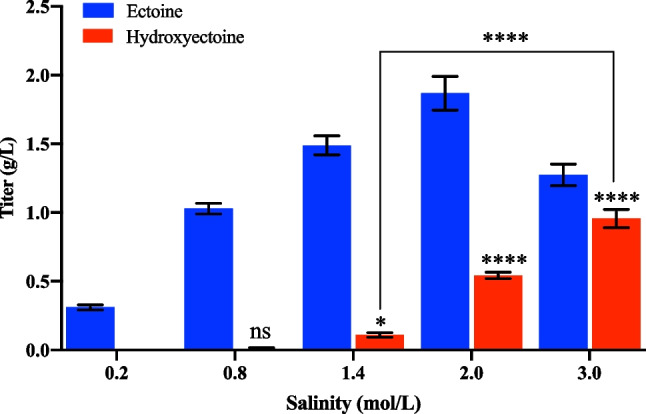
Hydroxyectoine production in *H. salifodinae* IM328 is positively correlated with the salinity of medium. The total titers of ectoine and hydroxyectoine were 0.31, 1.04, 1.60, 2.41, and 2.23 g/L at a salinity of 0.2, 0.8, 1.4, 2.0, and 3.0 mol/L NaCl, respectively. Ectoine and hydroxyectoine biosynthesis under different salinities were measured by HPLC. The error bars represent the standard deviations of the averages of three replications

Salinity has been shown to affect the production of ectoine and hydroxyectoine in halophiles (Bursy et al. 2007; Joghee and Jayaraman 2016; Seip et al. 2011; Tao et al. 2016). To examine the influence of salinity on the biosynthesis of hydroxyectoine in *H. salifodinae* IM328, we measured the hydroxyectoine production of *H. salifodinae* IM328 grown in media supplemented with 0.2, 0.8, 1.4, 2.0, and 3.0 mol/L NaCl, respectively. The hydroxyectoine production was positively correlated with the salinity. At salinity levels below 1.4 mol/L, almost no hydroxyectoine was detected in *H. salifodinae* IM328, which achieved a titer of 0.96 g/L hydroxyectoine at a NaCl concentration of 3.0 mol/L. *H. salifodinae* IM328 also produced ectoine, the titer of which was correlated with the salinity up to 2.0 mol/L NaCl. Ectoine production reached 1.87 g/L in conditions of 2.0 mol/L NaCl (Fig. 2).

### Comparative transcriptomic analysis of *H. salifodinae* IM328 grown under low-salt and high-salt conditions

**Fig. 3:**
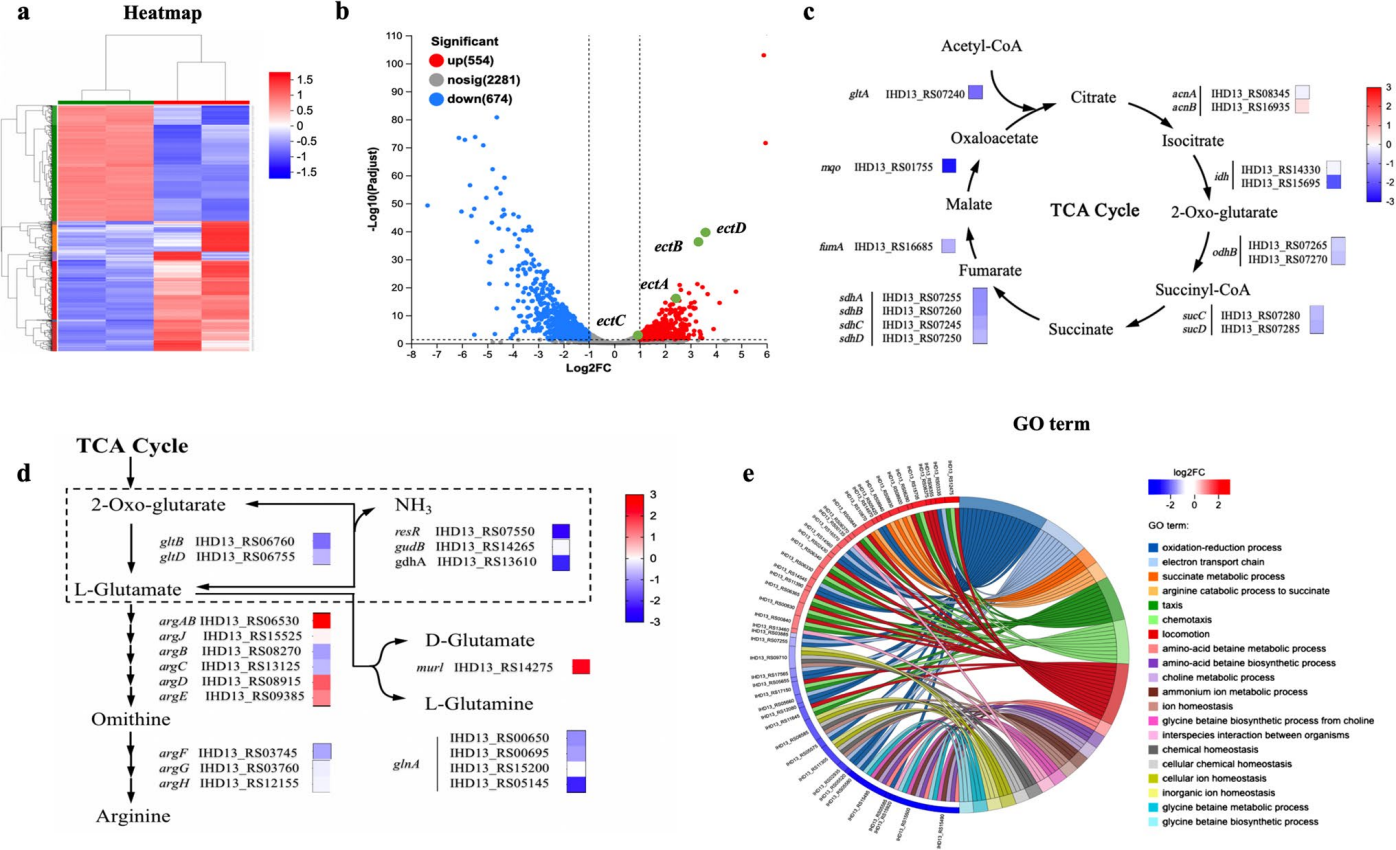
Comparative transcriptomic analysis suggests that the expression level of *ectD* and the availability of 2-oxoglutarate have profound influences on the hydroxyectoine biosynthesis in *H. salifodinae* IM328. **a** Heatmap analysis of gene expression profile of *H. salifodinae* IM328 grown under high-salt and low-salt conditions, respectively. The shades of colors represent the relative gene expression levels. **b** Volcano plot of the DEGs. Red dots indicate significantly upregulated genes, while blue dots indicate significantly downregulated genes. Green dots represent the key genes for hydroxyectoine biosynthesis. The |log_2_FC| > 1 and an adjusted *P* < 0.05 was used as threshold values. **c**, **d** DEGs related to 2-oxoglutarate metabolism. The shades of colors represent the relative gene expression levels. **e** The top 20 GO enrichment terms from differentially expressed genes (DEGs).

To uncover how hydroxyectoine biosynthesis responded to the increased salinity and find out the limitations on hydroxyectoine production under low-salt conditions, we performed a comparative transcriptomic analysis of *H. salifodinae* IM328 grown under low-salt and high-salt conditions. The gene expression profile was significantly altered when *H. salifodinae* IM328 growing in LB medium containing 0.2 mol/L NaCl was switched to LB medium containing 3.0 mol/L NaCl (Fig. 3a). *H. salifodinae* IM328 significantly upregulated the expression of hydroxyectoine biosynthesis genes (*ectA*, *ectB*, *ectC*, and *ectD*), especially the *ectD* gene encoding ectoine hydroxylase (Fig. 3b). Furthermore, the increased salinity resulted in the decreased expression of genes responsible for the tricarboxylic acid (TCA) cycle, a pivotal process for breaking down organic molecules to generate reducing power (FADH_2_ and NADH) and energy that organisms need to grow (Fig. 3c). Besides, the KEGG analysis revealed a decrease in the expression of genes directly associated with 2-oxoglutarate-related pathways (Fig. 3d). In addition, GO enrichment analysis of DEGs showed that the expression of genes involved in chemotaxis and locomotion increased significantly under high-salt conditions (Fig. 3e).

### Overexpression of *ectD* for lower-salt hydroxyectoine production

**Fig. 4:**
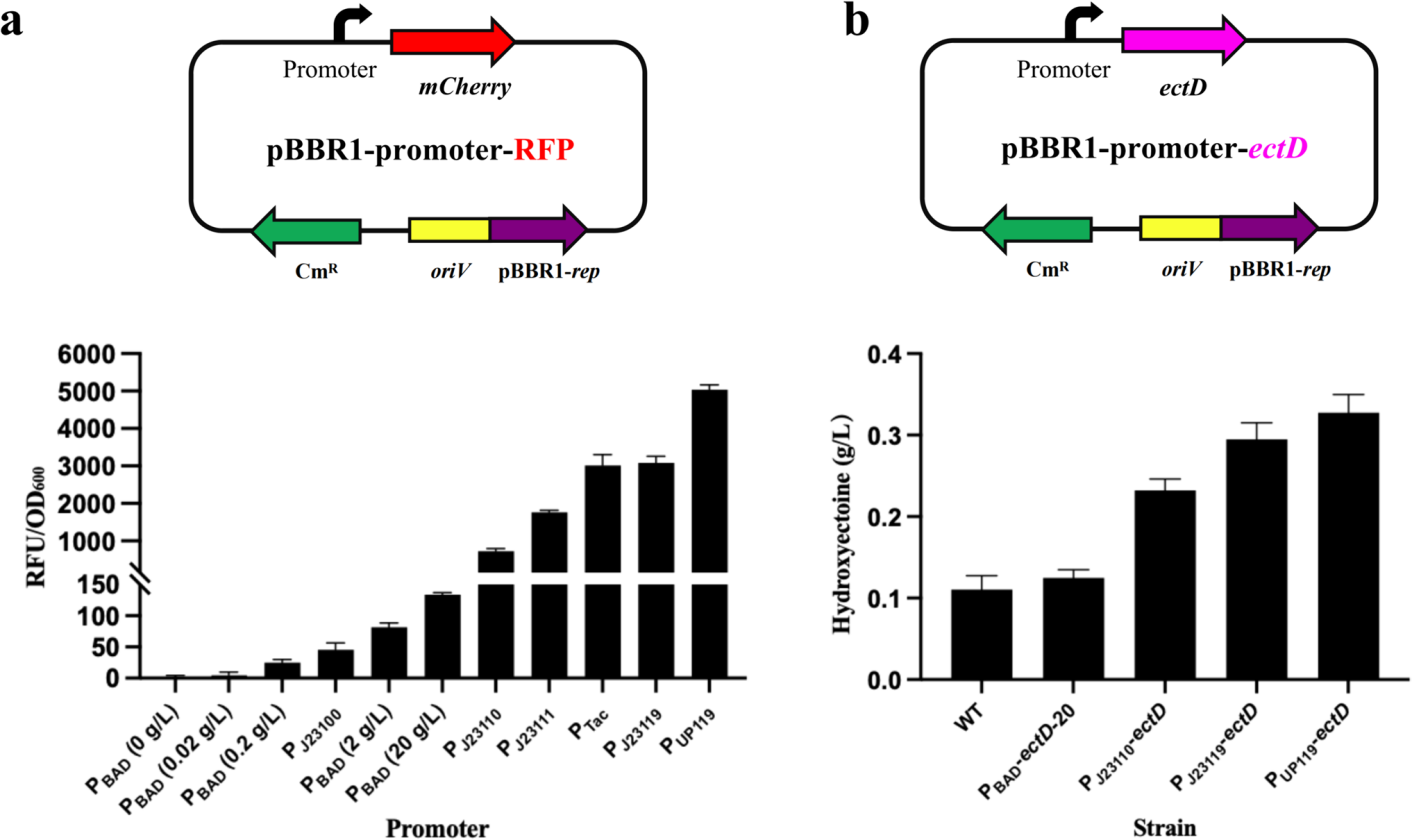
The hydroxyectoine production correlates with the expression level of *ectD*. **a** The strengths of a variety of promoters were assayed using a red fluorescent protein (RFP) reporter system in *H. salifodinae* IM328. The transcription of *mCherry* gene coding for the RFP was under the control of a specific promoter for each assay, and the promoter strength was determined by measuring the relative fluorescence intensity (RFU/OD_600_) of RFP. **b** The effect of the expression level of *ectD* on the production of hydroxyectoine. The engineered *H. salifodinae* strains (HS328-YNP08 to HS328-YNP11) were cultured in MME medium supplemented with 1.4 mol/L NaCl, a relatively low salinity. The error bars represent the standard deviations of the averages of three replications

To test whether the increased expression of *ectD* could improve the production of hydroxyectoine in *H. salifodinae* IM328 grown under lower-salt conditions, we need to express different amounts of *ectD*. Therefore, the *mCherry* gene driven by P_UP119_, P_J23119_, P_Tac_, P_J23111_, P_J23110_, P_J23100_, or P_BAD(*araC*)_ promoter was incorporated into the derivative plasmid of broad-host-range plasmid pBBR1MCS1 to examine the promoter strength, which was indicated by the relative fluorescence intensity. Among the promoters tested, the P_UP119_ promoter exhibited the strongest activity (Fig. 4a).

Subsequently, recombinant plasmids expressing *ectD* gene under the control of the P_UP119_, P_J23119_, P_J23110_, and P_BAD (*araC*)_ promoters were constructed and introduced into *H. salifodinae* IM328. Given that *H. salifodinae* IM328 grown with 1.4 mol/L NaCl produced much more ectoine than grown with 0.2 or 0.8 mol/L NaCl, the engineered *H. salifodinae* strains (HS328-YNP08 to HS328-YNP11) were cultured in MME medium supplemented with 1.4 mol/L NaCl. Though a three-fold higher production of hydroxyectoine was obtained by expressing *ectD* gene transcribed from the strongest P_UP119_ promoter, the titer of hydroxyectoine production (0.33 g/L) was still rather low (Fig. 4b).

### Prevention of the hydroxyectoine degradation by blocking the hydroxyectoine degradation pathway

**Fig. 5:**
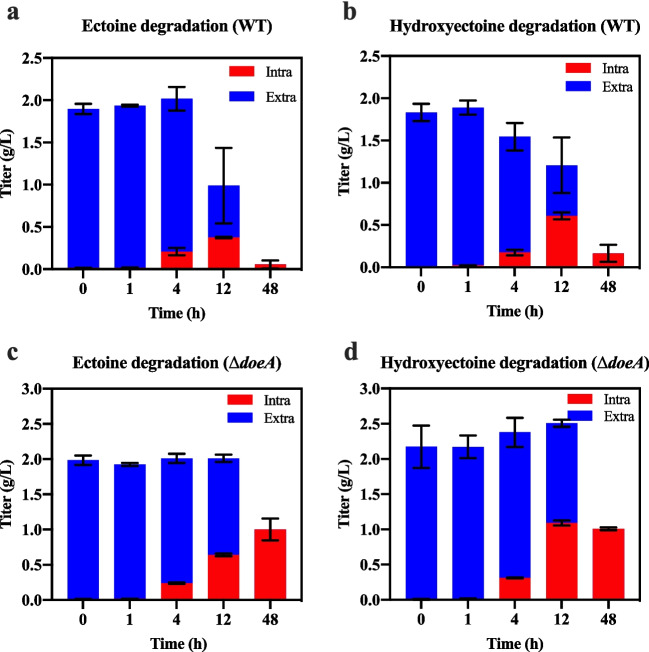
The deletion of *doeA* gene prevents ectoine and hydroxyectoine degradation to a large extent. The wild-type *H. salifodinae* IM328 and the *H. salifodinae* Δ*doeA* mutant (strain HS328-YNP12) were grown in 0.2 M MME media supplemented with 2 g/L ectoine or hydroxyectoine. **a**, **b** Nearly no detectable amounts of ectoine or hydroxyectoine remained in the wild-type *H. salifodinae* IM328 after 48-h incubation. **c**, **d** After incubating the *H. salifodinae* Δ*doeA* mutant (strain HS328-YNP12) for 48 h, 1 g/L of ectoine or hydroxyectoine were detected within the cells. Ectoine and hydroxyectoine were quantified by HPLC. The error bars represent the standard deviations of the averages of three replications. Intra, intracellular; Extra, extracellular

To examine if a low-salt environment triggers degradation of ectoine and hydroxyectoine, we added 2 g/L ectoine or hydroxyectoine to *H. salifodinae* IM328 cultures grown in low-salt conditions (0.2 mol/L NaCl). Initially, the intracellular ectoine or hydroxyectoine gradually increased with time, suggesting that the extracellular ectoine and hydroxyectoine were transported into *H. salifodinae* IM328 cells. After 48-h incubation with wild-type *H. salifodinae* IM328 cells, nearly no detectable amounts of ectoine or hydroxyectoine remained (Fig. 5a and b).

Our genome analysis of *H. salifodinae* IM328 revealed a *doeABXCD-eutBC* gene cluster that is potentially responsible for the degradation of ectoine/hydroxyectoine (Schulz et al. 2017; Schwibbert et al. 2011). Therefore, improved hydroxyectoine production could benefit from the deletion of ectoine/hydroxyectoine degradative genes. To delete the *doeABXCD-eutBC* gene cluster, we developed a genetic operating system based on the counterselectable marker *sacB* gene. Unfortunately, we failed to delete the whole *doeABXCD-eutBC* gene cluster from the *H. salifodinae* IM328 genome. As an alternative, we deleted the *doeA* gene, which is responsible for the first step of ectoine degradation in *Halomonas elongata* (Schwibbert et al. 2011). After incubating the *H. salifodinae* Δ*doeA* mutant (strain HS328-YNP12) with 2 g/L ectoine or hydroxyectoine for 48 h, 1 g/L of ectoine or hydroxyectoine remained within the cells (Fig. 5c and d). Although the deletion of *doeA* gene was unable to completely prevent the degradation of ectoine and hydroxyectoine, a substantially reduced degradation rate was achieved compared to the wild-type strain.

### Engineering of *H. salifodinae* IM328 for improved hydroxyectoine production under lower-salt conditions

**Fig. 6:**
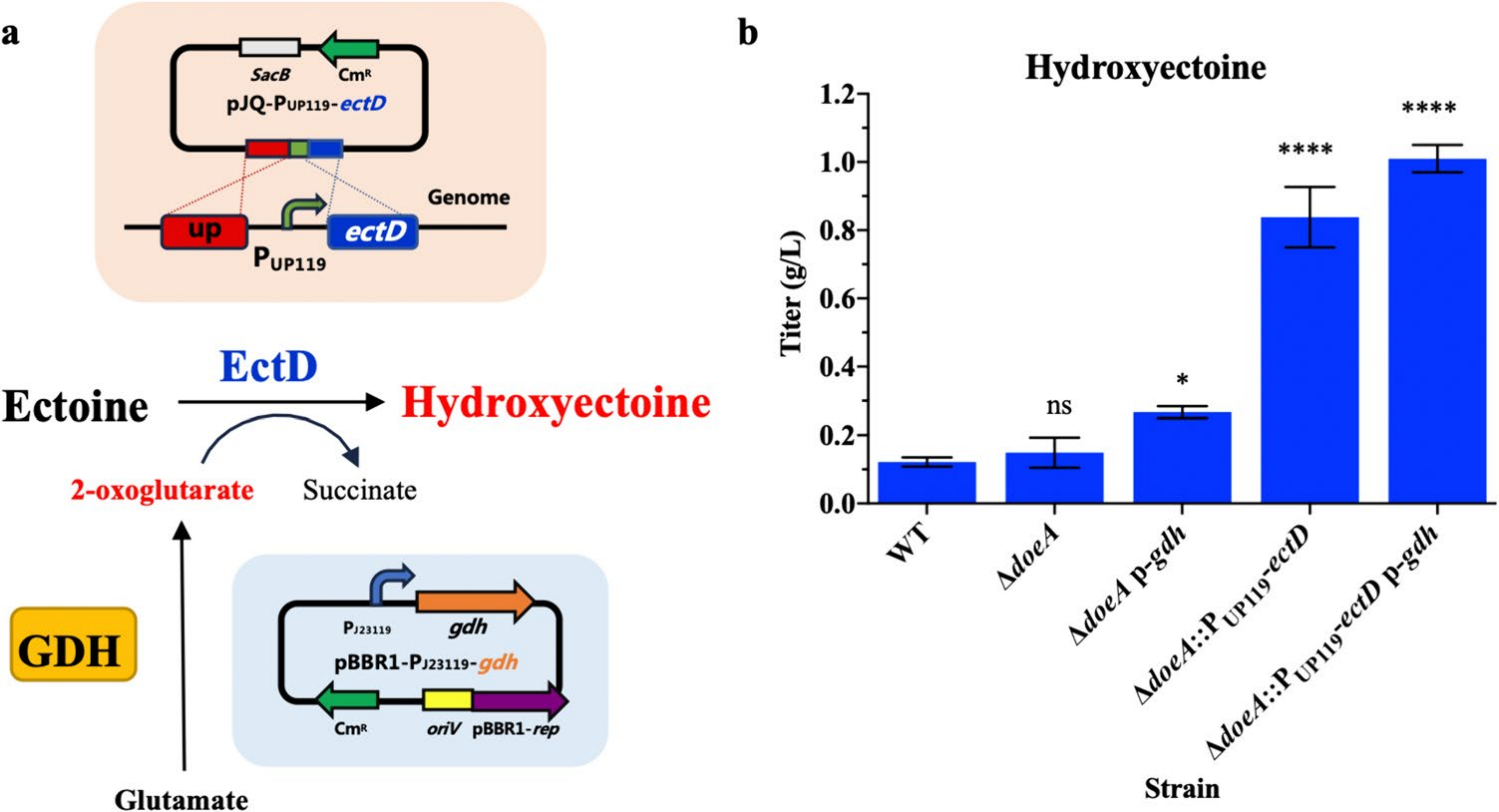
Engineering of *H. salifodinae* IM328 for enhanced production of hydroxyectoine. **a** Schematic representation of the strategy for optimizing the *H. salifodinae* IM328 chassis cells that are used to improve the hydroxyectoine biosynthesis under lower-salt conditions. **b** The engineered *H. salifodinae* Δ*doeA*::P_UP119_-*ectD* p-*gdh* (strain HS328-YNP15) by deleting the *doeA* gene and simultaneously overexpressing *ectD* and *gdh* genes produced 8.3-fold more hydroxyectoine than the wild-type strain under lower-salt conditions. The error bars represent the standard deviations of the averages of three replications

To improve the production of hydroxyectoine, *H. salifodinae* IM328 was engineered by deleting the ectoine/hydroxyectoine degradation pathway, enhancing the expression level of *ectD* and increasing the supply of 2-oxoglutarate (Fig. 6a). Given that glutamate can be easily used to make 2-oxoglutarate and is also a cheaper substrate than aspartate, we used MMD medium that contains glutamate in place of aspartate in MME. *H. salifodinae* Δ*doeA* mutant (strain HS328-YNP12) grown in lower-salt conditions accumulated more ectoine compared to the wild-type *H. salifodinae* IM328, suggesting the deletion of *doeA* gene partially prevented ectoine from entering its degradation pathway (Fig. 5a and c). However, the hydroxyectoine production showed no improvement (Fig. 6b).

To examine whether the increased expression of *ectD* can improve the production of hydroxyectoine, we overexpressed the *ectD* gene by replacing its native promoter with a strong constitutive P_UP119_ promoter in strain HS328-YNP12, generating the engineered *H. salifodinae* Δ*doeA*::P_UP119_-*ectD* (strain HS328-YNP13). After a 48-h shake flask fermentation under lower-salt conditions, we observed a significant improvement in hydroxyectoine production. The engineered *H. salifodinae* HS328-YNP13 produced 6.9-fold as much hydroxyectoine (0.84 g/L) as the wild-type strain.

Based on the comparative transcriptomic analysis, the availability of 2-oxoglutarate was another critical factor influencing hydroxyectoine production in *H. salifodinae* IM328 because it is a co-substrate for the ectoine hydroxylase EctD (Garcia-Estepa et al. 2006). However, the expression level of the *gdh* gene, which encodes glutamate dehydrogenase GDH that catalyzes the conversion of glutamate to 2-oxoglutarate (Johnson and Westlake 1972), was relatively low in *H. salifodinae* IM328 grown under low-salt conditions. It was less than half that of the expression level of the *rpoD* gene encoding the RNA polymerase sigma factor. To provide *H. salifodinae* strains HS328-YNP12 and HS328-YNP13 with sufficient 2-oxoglutarate, we overexpressed the *gdh* gene. The *H. salifodinae* Δ*doeA* p-*gdh* (strain HS328-YNP14) generated by mobilizing the pBBR1-P_J23119_-*gdh* plasmid into strain HS328-YNP12 produced 0.27 g/L hydroxyectoine, 1.45-fold higher than the wild-type strain (Fig. 6b). This result demonstrates that the increased supply of 2-oxoglutarate is able to enhance the biosynthesis of hydroxyectoine. Remarkably, by mobilizing the pBBR1-P_J23119_-*gdh* plasmid into strain HS328-YNP13, the engineered *H. salifodinae* Δ*doeA*::P_UP119_-*ectD* p-*gdh* (strain HS328-YNP15) finally produced 1.02 g/L hydroxyectoine in shake-flask culture, showing a 8.3-fold higher hydroxyectoine production than the wild-type strain (Fig. 6b).

To further test the performance of the engineered strain in continuous fermentation, we carried out the fed-batch fermentation of *H. salifodinae* HS328-YNP15 strain using a 5-L bioreactor filled with 2-L MMD medium. The ectoine/hydroxyectoine accumulation was positively correlated with the cell growth, with more hydroxyectoine synthesized at the later stage of fermentation. Without any detailed optimization of the fermentation process, the engineered strain HS328-YNP15 achieved a hydroxyectoine titer of 4.9 g/L after incubation for 60 h, representing a good performance for hydroxyectoine production under lower-salt conditions.

## Discussion

Increased salinity has been shown to stimulate the production of ectoine and hydroxyectoine in many microorganisms (Bursy et al. 2007; Joghee and Jayaraman 2016; Seip et al. 2011; Tao et al. 2016), indicating the fact of ectoine and hydroxyectoine as compatible solutes for organisms to resist hyperosmotic stress. We found that *H. salifodinae* IM328 isolated from a soda lake also accumulated ectoine and hydroxyectoine to cope with challenges caused by high salinity (Fig. 2). Although the production of ectoine first increased and then decreased with increasing salt concentration, hydroxyectoine was produced only under higher salinities and had a linear relationship with the salinity (Fig. 2). A similar phenomenon was also observed in moderate halophile *Virgibacillus halodenitrificans* PDB-F2 (Tao et al. 2016). These observations indicate that hydroxyectoine biosynthesis responds to a much higher salinity compared to ectoine biosynthesis, consistent with the fact that hydroxyectoine has a better protection performance than ectoine (Pastor et al. 2010).

Genes responsible for hydroxyectoine biosynthesis were significantly upregulated, especially the *ectD* gene encoding ectoine hydroxylase, to enhance the production of hydroxyectoine, supporting the halophilic cells to better withstand the hyperosmotic stress (Fig. 3b). It was found that the enzymatic activity of EctD was not significantly stimulated by increased salinity (Widderich et al. 2014a). Therefore, the increased synthesis level but not the specific activity of EctD were responsible for the improved hydroxyectoine production under high-salt conditions. Besides its role in the TCA cycle and intracellular nitrogen metabolism, 2-oxoglutarate plays a pivotal role in hydroxyectoine production as a substrate for ectoine hydroxylation (Chen et al. 2019; Ma et al. 2022; Reuter et al. 2010). Therefore, there is a competition for 2-oxoglutarate between the TCA cycle and the hydroxylation process. In response to these challenging circumstances, *H. salifodinae* IM328 primarily utilized 2-oxoglutarate for the synthesis of hydroxyectoine by reducing the metabolic flux into the TCA cycle under high-salt conditions (Fig. 3c). Therefore, the increased expression of *ectD* and the oversupply of 2-oxoglutarate contributed to the enhanced hydroxyectoine biosynthesis under high salt conditions to a large extent.

The P_UP119_, P_J23119_, P_Tac_, P_J23111_, P_J23110_, P_J23100_, and P_BAD (*araC*)_ promoters exhibited different activities (Fig. 4a), facilitating the precise regulation of gene expression in *H. salifodinae* IM328. The increased expression of *ectD* improved the hydroxyectoine production in conditions of 1.4 mol/L NaCl (Fig. 4b), indicating the importance of high EctD synthesis for enhanced hydroxyectoine biosynthesis under lower-salt conditions. However, the highest titer of hydroxyectoine production in the engineered strains grown with 1.4 mol/L NaCl (0.33 g/L hydroxyectoine) was still lower than that in wild with 3.0 mol/L NaCl (0.96 g/L hydroxyectoine), implying that the produced hydroxyectoine may be directed into the hydroxyectoine degradative pathway for biodegradation under lower-salt conditions.

Our ectoine and hydroxyectoine degradation assays suggest that an ectoine/hydroxyectoine degradation pathway exists in *H. salifodinae* IM328 to balance the ectoine and hydroxyectoine levels in response to changes in environmental salinities. Besides their excellent performance in hyperosmotic stress protection, the carbon- and nitrogen-rich ectoine and hydroxyectoine have been found as nutrients for halophiles (Vargas et al. 2006). Hence, *H. salifodinae* IM328 produces more ectoine and hydroxyectoine to combat the hyperosmotic pressure when environmental salinity rises, and utilizes them as nutrients for cell growth when environmental salinity falls. Like *Halomonas* *elongata* DSM 2581^T^, *H. salifodinae* IM328 has a *doeABXCD-eutBC* gene cluster that is potentially responsible for the degradation of ectoine/hydroxyectoine (Schwibbert et al. 2011), which is reasonable given that the two strains belong to the same genus *Halomonas*. In *H.* *elongata* DSM 2581^T^, DoeA has been proved as ectoine hydrolase catalyzing the first step of ectoine degradation (Schwibbert et al. 2011). Here, the deletion of *doeA* gene reduced the degradation rate of both ectoine and hydroxyectoine (Fig. 5c and d), indicating that DoeA is responsible for the degradation of both compounds in *H. salifodinae* IM328. Similar conclusion was obtained in another salt-tolerant bacteria *Ruegeria pomeroyi*, where EutD/EutE (homologue to DoeA and DoeB, respectively) complex has been proved to be able to degrade both ectoine and hydroxyectoine with EutD responsible for the hydrolyzation of the two compounds (Mais et al. 2020). However, the deletion of *doeA* gene was unable to completely prevent the degradation of ectoine and hydroxyectoine (Fig. 5c and d), implying that other pathways responsible for the degradation of ectoine and hydroxyectoine exist in *H. salifodinae* IM328.

Overexpression of glutamate dehydrogenase (GDH) that catalyzes the conversion of glutamate to 2-oxoglutarate enhanced the production of hydroxyectoine under lower-salt condition (Fig. 6b), proving that the availability of 2-oxoglutarate was exactly a critical factor influencing hydroxyectoine production in *H. salifodinae* IM328. Chen et al. reported that the supply of 2-oxoglutarate increased the production of hydroxyectoine in *Halomonas salina* BCRC 17875 until the 2-oxoglutarate concentration reached to 50 mM, while bacterial growth and hydroxyectoine production were both decreased when over 50 mM 2-oxoglutarate was added (Chen et al. 2019). Ma et al. also observed a similar phenomenon in the engineered *E. coli* strains that produce hydroxyectoine (Ma et al. 2022). By substituting the 2-oxoglutarate addition with auto-regulating intracellular 2-oxoglutarate pool based on quorum sensing, Ma et al. further improved the production of hydroxyectoine and no ectoine accumulation in the absence of osmotic stress (Ma et al. 2022). These results implied that more flexible 2-oxoglutarate supply might be necessary for efficient lower-salt production of hydroxyectoine in *H. salifodinae* IM328.

With a series of metabolic optimization, the final obtained *H. salifodinae* strain HS328-YNP15 (Δ*doeA*::P_UP119_-*ectD* p-*gdh*) produced 1.02 g/L hydroxyectoine in shake-flask culture, showing a 8.3-fold higher hydroxyectoine production than the wild-type strain (Fig. 6b). In the fed-batch fermentation, the engineered HS328-YNP15 strain further achieved a hydroxyectoine titer of 4.9 g/L. These results confirm that increased synthesis of EctD is pivotal to enhance the hydroxyectoine biosynthesis under lower-salt conditions. Furthermore, the knockout of *doeA* gene also contributes to an increased supply of ectoine towards hydroxylation, thereby further improving the production of hydroxyectoine. Moreover, the increased supply of co-substrate 2-oxoglutarate improved the production of hydroxyectoine as well.

Comparative transcriptomic analysis showed that *ectA*, *ectB*, and *ectC* were all upregulated in *H. salifodinae* IM328 in response to the high-salt stress. Therefore, it is feasible to improve the production of direct substrate ectoine by optimizing the expression of *ectA*, *ectB*, and *ectC* genes in *H. salifodinae* HS328-YNP15 strain. Besides, even if we overexpressed *ectD* and *gdh*, the ectoine precursor still existed at the end of fermentation. Prabhu et al. improved the conversion of ectoine to hydroxyectoine by expressing an ectoine hydroxylase from *Streptomyces chrysomallus* (ThpD) in *H. elongata*, though the *thpD* expression plasmid was unstable without selective pressure (Prabhu et al. 2004). Czech et al. tested the ability of different EctD to convert ectoine into 5-hydroxyectoine in *E. coli* and found that only EctD from *P. stutzeri* and *Nitrosopumilus maritimus* could convert almost all the provided ectoine into hydroxyectoine (Czech et al. 2016). Therefore, an alternative EctD may be required for *H. salifodinae* HS328-YNP15 strain to further enhance the conversion of ectoine to hydroxyectoine.

Altogether, this study showed that the ectoine/hydroxyectoine degradation, the expression level of *ectD*, and the supply of 2-oxoglutarate were three major influencing factors for the biosynthesis of hydroxyectoine. The engineered *H. salifodinae* HS328-YNP15 strain (Δ*doeA*::P_UP119_-*ectD* p-*gdh*) with these limiting factors eliminated produced 8.3-fold more hydroxyectoine than the wild-type strain when they were grown under lower-salt but alkaline conditions, and it finally achieved a hydroxyectoine titer of 4.9 g/L in a fed-batch fermentation. In comparison to the commonly used process employed by non-halophilic chassis cells such as *E. coli* and *C. glutamicum*, this work demonstrates advantages for the next-generation of open unsterile fermentation, which can effectively save energy and fresh water. When compared with the traditional “bacterial milking” process for hydroxyectoine production by halophiles like *H. elongata*, this work significantly mitigates the challenges associated with high-salt wastewater treatment. Therefore, the strategy employed in this study can be used for further developments on the economic viability of hydroxyectoine and other value-added chemical production from the engineered halophiles.

## Supplementary information

Below is the link to the electronic supplementary material.

Supplementary File 1 (DOCX 14.6 KB)

## Data Availability

The data that support the findings of this study are available on request from the corresponding author. The transcriptome sequencing data have been deposited in the NCBI Short Read Archive (SRA) under accession number PRJNA1018600.
